# Making the anaesthetised animal into a boundary object: an analysis of the 1875 Royal Commission on Vivisection

**DOI:** 10.1007/s40656-020-00344-9

**Published:** 2020-10-14

**Authors:** Tarquin Holmes, Carrie Friese

**Affiliations:** Sociology Department, London School of Economics and Political Science, London, UK

**Keywords:** Anaesthesia, Vivisection, Boundary objects, Social worlds

## Abstract

This paper explores how, at the 1875 Royal Commission on Vivisection, the anaesthetised animal was construed as a boundary object around which “cooperation without consensus” (Star, in: Esterbrook (ed) Computer supported cooperative work: cooperation or conflict? Springer, London, 1993) could form, serving the interests of both scientists and animals. Advocates of anaesthesia presented it as benevolently intervening between the scientific agent and animal patient. Such articulations of ‘ethical’ vivisection through anaesthesia were then mandated in the 1876 Cruelty to Animals Act, and thus have had significant downstream effects on the regulation of laboratory animals in Britain and beyond. Constructing this ‘consensus’ around the anaesthetised animal, however, required first excluding abolitionists and inhumane scientists, and secondly limiting the interests of experimental animals to the avoidance of pain through anaesthesia and euthanasia, thereby circumventing the issue of their possible interest in future life. This consensus also served to secure the interests of vivisecting scientists and to limit the influence of public opinion in the laboratory to administrative procedure and scheduled inspection. The focus on anaesthesia was connected with discussions of what supporting infrastructures were required to ensure proper ethical procedure was carried out by scientists. In contrast to the much studied polarisation in British society between pro- and antivivisectionists after 1876, we understand the 1875 Commission as a conflict amongst scientists themselves, while also being an intra-class conflict amongst the ruling class (French in Antivivisection and medical science in Victorian society, Princeton University Press, Princeton, 1975).

## Introduction

The Cruelty to Animals Act of 1876 marked a key turning point in the use of animals for laboratory science. It was the first legislation to regulate the use of animals for science, and the first piece of animal welfare legislation in Britain to specifically enact oversight of the educated classes’ use of animals. The Cruelty to Animals Act further comprised a stringent licensing system, which is still in use today, to govern how scientists use animals. Among the restrictions to which animal scientists became subject was that: “The animal must during the whole of the experiment be under the influence of some anaesthetic of sufficient power to prevent the animal feeling pain”. Further, any animal likely to experience pain upon recovery from the anaesthetic had to be killed beforehand.^1^

In this paper we argue that the anaesthetised animal represented a “boundary object” (Star and Griesemer 1989) through which stakeholders articulated and contested the morality of animal life and death in science. We trace how anaesthetics came to be viewed as a prophylactic against unacceptable abuses in the laboratory, addressing animal suffering whilst allowing for the continued practice of physiological experimentation. Focusing on the proceedings of the 1875 Royal Commission that preceded the Act, supplemented by related works by proponents and opponents of vivisection, we assess the competing interests and actors involved and seek to elucidate how the boundaries of the laboratory, of physiology and of class privilege were sustained through the deployment of anaesthesia.

Numerous analyses of late 19th century British controversies over vivisection focus on the fallout from the 1876 Act and the growing polarisation between supporters of science and antivivisectionists that characterised the decades up to the First World War. This polarisation has been variously interpreted as: a socio-cultural division between a professionalising and institutionally expanding medical science with extensive intellectual and political pretensions and a reactionary antivivisectionist movement fearful of the “cold, barren alienation of a future dominated by the imperatives of technique and expertise” (French 1975); a gender and class divide between the apparent callousness of the ‘smooth cool men of science’ and the politics of compassion espoused by feminists, socialists and other social reformers (Kean 1998); an emotional divide between a calculating scientific sympathy and an intuitionist common compassion (Boddice 2016); and a contestation over the boundaries of the human and corresponding modes of biopolitical governance (Murphy 2014).

The focus on polarization itself has more recently been critiqued. Shmuely (2017), for example, explores how empathy was institutionalized following the Commission and with the 1876 Cruelty to Animals Act through the tandem processes of professionalizing not only science but also government in Victorian Britain. She shows how science and law were here ‘co-produced” (Jasanoff 2004; Reardon 2001). This article develops the critique of polarization, but instead concentrates on the Royal Commission that preceded the Act, and on the construction of the anaesthetised animal as a “boundary object” (Star and Griesemer 1989) that allowed for “cooperation without consensus” (Star 1993). The means for cooperation was prerequisite for legislation to take place.^2^ Complicating the traditional science-antivivisection dichotomy, we thus explore the ways in which scientists themselves were conflicted about vivisection. We further argue that the 1876 Act represented as much a negotiation within the scientific community as it was between scientists and humanitarians.^3^

Boundary objects (Star 1988; Star and Griesemer 1989) are entities that exist at the junctures where different “social worlds” interact in an “arena” or an area of sustained and shared interest and concern. As a boundary object, we show how the anaesthetised animal was a focus for the anxieties of scientists regarding not only the nature of pain but also the relationship between life and death and the limits to trust in science. Clarke and Star (2008: 113) have defined the social worlds framework as focusing “on meaning-making amongst groups of actors—collectivities of various sorts—and on collective action—people ‘doing things together’ (Becker 1986) and working with shared objects, which in science and technology often include highly specialized tools and technologies (Clarke and Fujimura 1992; Star and Ruhleder 1996)”. If social worlds are held together by a shared technology, and vivisection was increasingly becoming a crucial technology for holding physiology together as a social world at the end of the 19th century in Britain, then scientists themselves were conflicted about their social world. Indeed, these types of conflicts are central to the social worlds concept, as Clarke and Star continue (2008: 11): “Over time, social worlds typically segment into multiple worlds, intersect with other worlds with which they share substantive/topic interests and commitments, and merge. If and when the number of social worlds becomes large and crisscrossed with conflicts … the whole is analysed as an arena. An arena, then, is composed of multiple worlds organized ecologically around issues of mutual concern and commitment to action”. We argue that combining the technology of anaesthesia with vivisection allowed for scientists to persist as a social world, to continue to be held together as opposed to split. This required separating out ‘immoral’ scientists as a distinct “subworld” or “segment” composed of “mavericks” in order to maintain legitimation (Clarke and Star 2008: 118–119).

Scientists as a social world in Victorian Britain were overdetermined by class conflicts and anxieties, particularly given that all other regulations of animal treatment implemented during the 19th century were directed at controlling the working class. British scientists had an unsettled status within the British elite following mid-19th century professionalisation and secularisation, a process which Turner (1978) cites as undermining the old alliance between science and natural theology, leading to conflicts with the clergy over epistemic authority regarding cosmological and evolutionary questions. The growth in paid scientific positions also enabled an increasing influx of middling and working class men, “diluting” the profession’s former aristocratic pedigree.^4^ These changes should not, however, obscure the fact that scientific education and training in late 19th century Britain remained largely the reserve of privileged white men, and class consciousness within the scientific community was heavily shaped by elitist paternalism and belief in entitlement to freedom of enterprise and enquiry. We shall concentrate on the threat posed by charges of physiological cruelty to these traditional class privileges, specifically the claim that vivisectors, through practicing cruelty, suffered a loss of sympathetic inhibition against violence—‘demoralisation’—believed to manifest in the form of ‘blunted feelings’. By claiming that vivisection was antithetical to the maintenance of a genteel and moral character, antivivisectionists sought to undercut physiologists’ claims to class-based privileges of freedom from surveillance and regulation.

We seek to show how anaesthesia at the 1875 Royal Commission played a central role in defending and securing vivisection through not only protecting laboratory animals from pain but, by intervening between agent and patient, also protecting laboratory scientists from demoralisation. Through replacing pain and motion with oblivious stillness, anaesthetics helped partially and momentarily mend the schism over vivisection both amongst scientists and with humanitarians. It also, however, became apparent at the Commission that scientists could not be trusted to self-regulate, resulting in a failure of trust which legislation and supervision were required to compensate for. This imposition of external regulations on animal experimentation was regarded as an insulting intrusion by many scientists, even those who had little to do with experimental physiology, whilst also failing to satisfy those antivivisectionists who favoured abolishing vivisection. But it did introduce a new social world to the arena of vivisection: legislators and regulators. Thus the 1876 Act came to exacerbate divisions between life scientists and antivivisectionists even as it established a legislative and regulatory ‘consensus’.

Much of the earlier historical and social science literature on animals and science, as well as its regulation, has been organized according to the notion of a divide between anti-vivisection groups and scientists (Birke, Arluke and Michael 2007). Recent social science and humanities scholarship has troubled this divide through research that focuses on the contemporary moment, emphasizing that it is important to better understand how scientists themselves think about and respond to the welfare of animals used in experiments (Davies 2012; Hobson-West 2012; Sharp 2019) and to the debates over their use in research (Birke et al. 2007). Social scientists have examined how scientists and other laboratory workers respond to public debates regarding animal welfare in science (Hobson-West 2012; Michael and Birke 1994a, b; Hobson-West and Davies 2018). Hobson-West (2009, 2012) finds that scientists working in Britain find regulation to serve a supportive and legitimating role in their work. Shmuely’s (2017) historical research shows that focusing on divisions between antivivisection and science belies a more complex historical narrative as well. This paper contributes to this scholarship by showing that the social world of science was not unified at the 1875 Commission either, and the anaesthised animal was meant to resolve this as a boundary object. This worked in part because anaesthesia was considered a deathlike state, and death itself was not deemed a problem—something that persists in British regulation today.

## Humanitarian sentiments and anti-animal cruelty legislation in Britain before 1875

Vivisection first rose to prominence in Britain during the 17th century, when it was employed by William Harvey to investigate the circulation of blood, and would come to play a distinctive role in public demonstrations at the early Royal Society. From the beginning, concerns were raised that vivisection was cruel, with tensions evident between desire for physiological knowledge and recognition of animal pain. Guerrini (1989) discusses the case of Robert Hooke, who in 1664 carried out an experiment at the Royal Society whereby a dog was respirated by bellows whilst its chest was opened to expose its heart. The experiment was designed to demonstrate the necessary connection between respiration and the heart, which was evidenced by the fact the animals’ heartbeat became irregular if the bellows stopped (Guerrini 1989: 400–402). Despite the experiment’s success, the animal’s suffering nevertheless weighed heavily on Hooke’s conscience. Writing to Robert Boyle, he remarked that “I shall hardly be induced to make any further trials of this kind, *because of the torture of the creature*”, going on to state that “the enquiry would be very noble, *if we could find any way so to stupify the creature*, as that it might not be sensible, *which I fear there is hardly any opiate will perform*” (Ibid: 401; our emphasis).^5^

For Hooke, the tension between the concern to advance physiological knowledge and caring about animal suffering was very much real. An opiate potent enough to alleviate vivisectional pain might have allowed for a compromise, with the stupefied animal serving as a boundary object at the junctures of the interests of science and those of the animals. But in the absence of such a narcotic, Hooke eventually felt compelled to resume vivisecting. Following suggestions that the lungs’ movement, not the air itself, imparted motion to the heart, he carried out a second experiment in 1667 showing that a dog’s heart would still beat even if the lungs were fully inflated and rendered motionless (Ibid: 400–402). Despite Hooke’s concerns about animal suffering, his commitment to advancing physiological knowledge ultimately won out.

Physiological knowledge did not, however, always triumph at the animals’ expense. A growing humanitarian critique of animal mistreatment in the later 17th century, motivated both by religious arguments that cruelty was sacrilegious and secular arguments taking direct issue with animal pain, led in Britain to increasing disapproval of practices such as vivisection (Thomas (1984: 154), which went into relative decline after 1670. Although Guerrini (1989: 405–407) cites proximate factors such as funding shortages and declining participation as more immediate causes of the end of public vivisections at the Royal Society, she acknowledges that a more conservative cultural atmosphere also likely discouraged attempts to resurrect the practice. Whilst vivisection-based research continued to be carried out in 18th century Britain, e.g. Stephen Hales’ 1730s experiments on blood pressure (Meli 2013: 219–221) and John Hunter’s 1780s study of aneurysms (Murley 1984; Slagle 2011), this work was mainly carried out in private accommodation out the sight of prying eyes.

British public sentiment against cruelty to animals became increasingly entrenched during the 18th century, being attacked in its various manifestations by such prominent public intellectuals and artists as Alexander Pope (1831 [1713]), William Hogarth (Ireland and Nichols 1883 [1751]), William Cowper (1899 [1785]) and Jeremy Bentham (1789).^6^ Vivisection, due to its private and privileged character, was at first rarely a direct target of criticism—Samuel Johnson’s 1758 attack on the “inferior professors of medical knowledge” and their pursuit of knowledge through “knives, fire and poison” being a notable exception to this rule (Wiltshire 1991: 128–130). However, as antipathy towards animal cruelty began to take on legislative and legalistic forms towards the turn of the 19th century, greater scrutiny began to be paid to both public and private sites of abuse. Slaughterhouses and cattle markets were already coming under regulation and surveillance in the 1780s (Thomas 1984: 178), and Parliamentary bills against bull-baiting were tabled as early as 1800 (Turner 1980: 15–29). The first successful general piece of British parliamentary legislation was the Cruel and Improper Treatment of Cattle Act of 1822, commonly known as Martin’s Act after its proponent, the MP Richard Martin. Initially limited to domestic livestock, these protections were then extended through the 1835 Cruelty to Animals Act, which banned the keeping of animals “whether of domestic or wild Nature or Kind” for the purposes of fighting, running or baiting them and clarified that previously instituted protections to livestock also applied to “any other… domestic animal”.^7^ Both Acts were replaced by the 1849 Act for the more Effectual Prevention of Cruelty to Animals, which specifically prohibited that “any person shall cruelly beat, ill-treat, over-drive, abuse, or torture, or cause or procure to be cruelly beaten, ill-treated, over-driven, abused, or tortured any [domestic] animal”.^8^ The animal in pain thus became a juncture at which human interests in exploiting animals had now to be reconciled with perceived animal interests and the interests of those who cared about them.

Animal protectionists targeted vivisection as early as 1824, when Martin condemned in Parliament the demonstrations performed in London by the visiting French physiologist Francois Magendie (Olmsted 1944: 134–143). But British scientists led by Charles Bell were able to redirect humanitarian scrutiny by characterising cruelty as peculiar to Continental science and their own work as by contrast compassionate and not reliant on vivisection (Berkowitz 2015: 145–146). Implicit in Bell and company’s claim was the argument that, as respectable members of the educated and gentlemanly elite, they should be trusted to treat animals in a genteel, gentle manner. This reflected a broader class dynamic in how animal cruelty was perceived and articulated in 18th and 19th century Britain—cruelty towards animals was linked to the working class in Britain (Ritvo 1987: 125–166) and non-whites in the colonies (Chakrabarti 2010). The upper and educated classes were deemed more refined and robust in sentiments and therefore more trustworthy. The imposition of these class boundaries on the policing of animal cruelty would contribute to vivisection being one of the last areas of animal use to become regulated in Britain. Science as a social world was thus overdetermined by its class status.

The use of vivisection by educated and upper class scientists did, however, raise uncomfortable questions. Cruelty was widely regarded a mark of deficiency in moral character, a failing thought endemic among “the lower orders”. French (1975: 296) notes that the elites justified their privilege by claiming to educate the poor and feckless by moral example, and so civilise their vicious inclinations. Elite failure to edify the masses, 19th century British moralists reasoned, threatened unrest and ultimately class war, as had occurred in France during the Revolution. It was for this reason that cruelty, “the worst of vices”, was regarded by antivivisectionists as “most retributive to mankind… when perpetrated by the refined and educated” (Jesse 1875: x). Cruelty was also, with the rise of evolutionary theory, increasingly identified with biocultural primitiveness and the ‘animal’ in human nature (Boddice 2016). Darwin (2004 [1871]: 147) himself maintained that “humanity to the lower animals seems to be one of the latest moral acquisitions. It is apparently unfelt by savages, except towards their pets”. If cruelty to animals was the preserve of reprobates and savages, then vivisection, widely believed cruel, appeared to undermine pretensions to moral refinement made by upper class scientists and suggest the possibility of their demoralisation. Vivisection thus raised tensions regarding the class boundaries underlying British animal protection regimes, arousing fears of moral degeneration within the ruling classes. The role of vivisection in the social world of British physiologists was thus a debate not simply between scientists and humanitarians, but internal to the social world of science itself, as a profession predominantly pursued by members of the upper and educated classes.

## 1824–1874: a half-century of disciplinary growth and rising humanitarian concerns

The contention by Bell and other early 19th century British scientists that they, unlike their French counterparts, were compassionate towards animals and reluctant to vivisect was ultimately self-serving and glossed over the fact that many of them, Bell included, did on occasion practice vivisection. Assertions of a stark divide between British and continental physiology did however contain a grain of truth. British scientists remained largely wedded to the idea of inferring function from structure, privileging anatomy over physiology and dissection over vivisection, which tended to be employed sparingly in order to confirm anatomical theory (Berkowitz 2014: 7). In sharp contrast, French physiologists, led by Magendie, increasingly rejected this style of investigative reasoning in favour of a highly empirical experimentalism where function was determined through observing the consequences of targeted interventions in the living animal body (Stahnisch 2009). A valorisation of vivisection as a method of scientific investigation was furthermore central to the efforts of early 19th century Continental physiologists to establish disciplinary autonomy from anatomy, to which physiology had been institutionally and methodologically subordinate (Lemoine and Pradeu 2018: 236).

Alongside France, Germany was the other great centre for the discipline’s development and the foremost in terms of its institutionalisation, with nearly every university by 1860 having created a separate chair for physiology in its medical faculty, a mere 49 years after the first was established at Breslau (Kremer 2009: 344).

In Britain, it was not until 1874, the year before the Royal Commission, that the first chair for physiology was established: John Burdon-Sanderson’s professorship at University College London (Ibid). Burdon-Sanderson himself maintained that physiology had progressed at a similar pace in Britain as elsewhere, asserting that “all that has been done lately is to organize schools of physiology in a more complete manner than they were organized before” (Report 1876: 115). However, there was a clear recognition among both physiologists and antivivisectionists that institutionalisation constituted a turning point. Combined with the ongoing expansion of university-based scientific training, this would entail an increased utilisation of vivisection in Britain. This was recognised by the 1875 Committee, who remarked that:

It is indeed the expectation of those most conversant with the subject, that physiological investigations will more and more take place in connection with public institutions, that new chairs will from time to time be founded, and that an organised system of instruction in physiology will speedily become an important feature in scientific education. It is evident, therefore, that the number of experiments at present performed upon living animals can by no means be regarded as the limit of the number which we are called upon to include in our consideration, but that on the contrary we must assume that the experimental method is being rapidly developed (Ibid: viii).

Vivisection was thus no longer a marginal technology in the social world of British science, but was rapidly becoming central.

The institutionalisation and expansion of physiology entailed growth in associated infrastructure, two important forms of which were textbooks and buildings. These were both sites of controversy in the lead-up to the Royal Commission. The publication of Klein, Burdon-Sanderson, Foster and Brunton’s 1873 *Handbook for the Physiological Laboratory* provoked an uproar due its description of apparently painful animal experiments without explicit instruction to employ anaesthetics (French 1975: 47–50). Concerns were raised that novices might use the book to carry out unsupervised private experiments, fears apparently later confirmed at the Commission by John Anthony, who claimed reliable knowledge of “juveniles” (i.e., young medical practitioners) vivisecting. He cited the assistance given by “certain books on these subjects” that “very carefully directed… how you are to cut the creature” (Report 1876: 135). Concerns were likewise raised at the Commission about buildings, particularly Edinburgh University’s 1874 application to add a physiology laboratory with designated space for keeping animals (Jesse 1875: 26–27). Again, young medical practitioners were a focus for concern, with some senior scientific witnesses suggesting the expansion of facilities and a decrease in supervision would allow “blockheads” (Samuel Haughton) to inflict unnecessary pain on animals (Report 1876: 104). If infrastructures can be understood “as frozen discourses that form avenues between social worlds and into arenas and larger structures” (Clarke and Star 2008: 115), then textbooks and buildings were avenues through which physiology and vivisection were moving from the margins to the center of science as a social world.

The growth in physiology departments, student numbers and related infrastructure all pointed to what many considered an inevitable increase in the number of animals being vivisected. This led to growing concerns among humanitarians, many of whom, including later antivivisectionist luminary Frances Power Cobbe, had already participated in campaigns against Continental vivisection.^9^ This heightening conflict between an expanding and institutionalising physiology reliant on vivisection and a popular humanitarian sentiment repulsed by animal cruelty would, as an arena, force itself before the law in December 1874. Then, RSPCA Secretary John Colam attempted to prosecute four Norwich doctors for aiding and abetting the French physiologist Eugene Magnan in performing a demonstration of the effects of intravenous alcohol and absinthe on two dogs at the August 1874 meeting of the British Medical Association (Anon 1874: 751–754). Although the doctors were ultimately acquitted on grounds of insufficient proof of involvement, the magistrates disallowed reimbursements of defence costs. They thereby indicated agreement that it was a worthy case to prosecute and that doctors and scientists’ class privileges should not preclude them from standing trial for alleged animal cruelty (French 1975: 55–60). The arena of vivisection was thus expanding to also include the social world of physicians in close relationship to physiologists as well as the social world of legislators. This is the arena that the 1875 Royal Commission became part of, and that the anaesthetized animal came to be created as a boundary object to mediate.

## The 1875 Royal Commission: an overview

In May 1875 antivivisectionists led by Frances Power Cobbe successfully lobbied Lord Henniker to table a bill restricting animal experimentation and enforcing inspections. In response, the MP Lyon Playfair proposed a counter-bill in defence of medical and scientific interests that nonetheless included strong restrictions on animal pain, including a mandated use of anaesthetics except where permitted under special license. After both bills failed to win enough support (in part due to being poorly drafted), the need for a Royal Commission on vivisection became apparent, and its announcement received a “general welcome” (Ibid: 69–79).

In selecting members for the Committee for the Royal Commission, Home Secretary Richard Cross attempted to achieve “a balance of scientific expertise, practical medical experience, judicial wisdom, and humanitarian zeal”, restricting partisan participation and favouring candidates who were ideologically flexible (Ibid: 112–113). This selection process did bias towards *some* form of legislative compromise emerging but did still leave considerable room for disagreement.

The Committee was chaired by Lord Cardwell, former Secretary of State for War and a vice president of the RSPCA (which opposed needlessly painful experimentation but not vivisection per se).^10^ The politicians Sir John Karslake, Lord Winmarleigh and W.E. Forster were selected to represent a relatively uncommitted ‘middle ground’, although Forster, also an RSPCA vice president, opposed painful experimentation and had previously clashed with Huxley on the subject. The selection of John E. Erichsen, Professor of Surgery at UCL, was also somewhat controversial, as he was appointed purportedly to represent antivivisectionist medical opinion, but during the Commission generally gave qualified support to vivisection and defended his past record of animal experimentation (Jesse 1875: 111–114; French 1975: 92–96).^11^ Representing antivivisection and science on the Committee were, respectively, Richard Holt Hutton, editor of the *Spectator*, and Thomas Henry Huxley, famed defender of Darwinism. Hutton was the main advocate of strongly restrictive legislation on the Committee but whilst favouring an end to vivisection recognised that an immediate ban was not politically realistic (French 1975: 51–55). Huxley sought to defend scientific autonomy and limit the scope of legislation, and could be understood as the representative or “entrepreneur” (Clarke and Star 2008: 118) of physiology in the social world of science. But prior to the Commission he had in private correspondence with Charles Darwin stated that “as a matter of right and justice in the first place and secondly as the best method of taking the wind out of the enemy’s sails”, it was best to advocate for humane but reasonable legislation (Huxley 1908: 167). Overall, whilst the Committee largely agreed that experimental pain was problematic, there was no inbuilt majority in favour of particular legislation. Instead, a consensus favouring specific legislation was contingently constructed in the course of the Commission, as based on the evidence of witnesses and the interpretations of the Committee, with Hutton and Huxley jostling for support from their colleagues.

To establish a working consensus in favour of restrictive legislation, the Committee had to reconcile two opposing and incompatible “positions” (Clarke 2005) on animal experimentation. The first we shall refer to as the *laissez*-*faire* faction, an anti-restrictionist position favouring scientific autonomy. Some physiologists believed in the inherent value of using vivisection to advance abstract scientific knowledge and rejected any form of practical restrictions (French 1975: 217 & 306). Whilst witnesses at the Commission generally did not explicitly espouse this position, its rationale does appear to animate Edinburgh Anatomy Professor William Turner’s warning that “when legislative checks are enacted, the progress of discovery in science is likely to be impeded” (Report 1876: 161). Other scientists rejected legislation as an interference in individual liberty and maintained that the scientific community was better left to self-regulate. Burdon-Sanderson thus declared that “in England individual action in all things is the national tendency, and it is just the same in science as in other things”. He went on to assert that “legislation is certainly not necessary, because there is no influence which legislation could exercise of a beneficial character upon [scientific] institutions, which is not already exercised by the organization under which they are placed”, namely “the direction of committees… containing men of position in society… [or] the immediate direction of educated and responsible men” (Ibid: 122). That this claimed right to individual action was tied to assumed class privileges is made evident by surgeon and Privy Council medical officer John Simon’s protestation that “You are proposing that physiologists shall be treated as a *dangerous class*, that they shall be licensed and regulated like *publicans and prostitutes*” (Ibid: 75; our emphasis). Simon makes clear that when supporters of *laissez*-*faire* appealed to scientists’ rights to liberty they did not presume these to be rights owed to them simply as citizens of Britain but rather as members of the ruling class and educated elite who, unlike publicans, prostitutes and the ‘mob’ (see below), should be granted enough trust to carry out their activities without supervision. The social world of science was thus overdetermined by the class relations of Victorian Britain.

Diametrically opposed to *laissez*-*faire* was abolitionism, represented among witnesses at the Commission by George R. Jesse, founder and honorary secretary of the recently established Society for the Abolition of Vivisection, and his supporter the MP James Maden Holt. Staking out the abolitionist position, Jesse denounced both justifications of vivisection as advancing knowledge and as improving healthcare, upholding that “The plea of knowledge to be obtained is no excuse; the good of humanity is no excuse. We have more knowledge already than we use for good”. Moreover, he expressed moralistic scepticism regarding the rationale animating scientific medicine, maintaining that “Folly, vice, ignorance, dirt, and selfishness create disease”, and therefore “To torture animals to escape the natural penalty of viciousness, is… striking at the effect and not the cause”. Allying with the views of many hygienists and cultural conservatives, Jesse maintained that what was necessary to combat disease was “moral reform and the diffusion of [existing] knowledge”. “Even if”, he further clarified, “any discoveries in knowledge had been extorted and wrung out by torture… such discoveries are gained by injustice, and being so, will ultimately prove… worse than profitless, to the human race” (Jesse 1875: 123–126). Scientists would do better, Jesse implied, to try to educate and edify the dangerous classes rather than succumbing to vice in a vain effort to overturn Providence. The social world of antivivisection was thus overdetermined by the class relations of Victorian Britain as well, and the arena of vivisection can thus be seen as an intra-class conflict amongst those in power regarding the future of national identity.

Overall, the Committee sympathised with neither the abolitionist nor *laissez*-*faire* positions. Jesse, in his own view, “received little countenance or encouragement” from any Commissioner apart from Winmarleigh (Ibid: vii). As we shall see, Klein’s testimony did much to discredit the *laissez*-*faire* claim that scientists could be left to self-regulate. Neither the positions that common morality should superimpose itself on scientific practice nor that scientific practice should be free from external scrutiny were deemed acceptable. But rejecting these absolutist positions required constructing an alternative site of consensus based on agreements as to what constituted acceptable and unacceptable practices and what was to be deemed necessary and unnecessary experiment. The anaesthetised animal became a boundary object allowing for an apparent compromise between the needs of science and the concerns for the suffering animal, a compromise alluded to but not available to Hooke in 1667. The boundary object thus became a site of compromise within the social world of science as well.

## Anaesthesia as a Purported Panacea: making a boundary object

Introducing their 1875 Report, the Committee claimed that “inflicting any pain that was not absolutely necessary upon any animal” was broadly regarded as unacceptable in the British scientific community (Report 1876: x). The Committee concluded that there should be no toleration for “purposeless cruelty” (Ibid: 20–21), but that pain induced as part of “necessary experiments”^12^ could be justified if otherwise unavoidable. Yet this begged the question what constituted a ‘necessary experiment’.

The Committee sought to answer this question in part by identifying aspects of physiological research where there was potential for ‘purposeless cruelty’. On the advice of Alfred Taylor, Lecturer on Medical Jurisprudence and Toxicology at Guy’s Hospital, they identified four areas of concern—“experiments made in excessive numbers”^13^; “experiments made to establish what has been already proved”; “experiments made where a man has been desirous of bringing himself forward, or trying a new thing merely for the sake of a little notoriety”; and “experiments attended with great pain, and defeating the very object in view” (Ibid: xiv; see 55–61 for Taylor’s testimony). Ultimately, the 1876 Act would principally address these four areas.

In terms of the first three concerns, excessive and unnecessary experimentation was restricted by limiting replication to confirmatory tests “absolutely necessary for the effectual advancement of such knowledge” (1876 Act: Section 3). A licensing system, including workplace inspections, furthermore criminalised dilettante experimentation without oversight and sought to prevent experimental excesses by requiring that physiologists report and justify their practices to the licensing body. Experiments furthermore needed to be justified in utilitarian terms as advancing the discovery of “physiological knowledge or of knowledge which will be useful for saving or prolonging life or alleviating suffering” (Ibid).

Anaesthetics were to be the main means for tackling pain, mandated except where “insensibility cannot be produced without necessarily frustrating the object of such experiments” (Ibid). The rationale for anaesthetics was heavily dependent on the utilitarian claim that they prevented purposeless cruelty. The Committee went so far as to say that “The whole subject of experiments upon living animals has been, or at least ought to have been, relieved of the greater part of its difficulty by the discovery of anaesthetics” (Report 1876: xi.). In former times a committee would have had to “weigh in the one scale the infliction of great and perhaps protracted suffering, and in the other the sacrifice of knowledge, most important to mankind” (Ibid: xiii). Anaesthetics, by facilitating that “the great majority of experiments can be rendered very nearly, if not entirely, painless” (Ibid: xi), relieved the experimenter of many of their most pressing ethical concerns. Commissioner Erichsen thus spoke of “a new era as having been introduced in experimentation on animals since the introduction of anæsthetics” (Ibid: 3). Anaesthesia appeared to offer a technological solution to Hooke’s 1667 quandary, Darwin going as far as to suggest that vivisection under anaesthetics should be referred to as ‘anaes-section’ to emphasise its complete ethical removal from the physiological cruelties of the pre-anaesthetic age (Boddice 2016: 93).

If vivisection was moving from the margins to the centre of British science as a social world in a manner that raised concerns, anaesthesia offered a possible corrective. By tightly binding vivisection with anaesthesia, the social world of British science could be held together. It was also hoped that this would provide a compromise for the arena forming around vivisection—and the intra-class concerns that were being articulated through the arena.

## Building and contesting the infrastructures of anaesthesia

Whilst the ethical benefits of anaesthesia were much touted, its practical advantages had troubling consequences for what counted as humane animal treatment. Anaesthetics were cited at the Commission by George Humphry as one of the two major factors leading to an increase in vivisection, the other being an increase in scientific personnel. Humphry attributed this influence to the fact that “experiments being possible under anæsthesia renders physiologists so much less reluctant to undertake them” (Ibid: 33). He saw this lesser reluctance as positively removing barriers to experimentation. But for antivivisectionists this lesser reluctance was evidence of a loss of moral concern for animal life and wellbeing. For Jesse, his opposition to vivisection was borne out of “intimacy and close friendship with animals” (1875: 2). The mere removal of animal pain did not resolve the problem of animal experimentation more generally.

But the most damaging attack on the credibility of anaesthetics came from within science itself, by George Hoggan, in February 1875, a mere five months prior to the start of the Royal Commission. Hoggan had previously spent four months working in the esteemed French physiologist Claude Bernard’s Paris laboratory. With public unease about vivisection growing following the December 1874 Norwich prosecutions, Hoggan ventured to express his own reservations based on his experiences on the Continent. In a letter to *The Morning Post*, Hoggan reported witnessing numerous abuses, including the reuse of vivisected animals for student anatomy lessons whilst still alive and the slapping of animals in response to cries of pain. Anaesthetics, he alleged, were “little depended on” as “They alter too much the normal conditions of life to give accurate results”, and were in truth “far more efficacious in lulling public feeling towards the vivisectors than pain in the vivisected”.^14^ Rather than a panacea for experimental pain, Hoggan was instead “inclined to look upon anaesthetics as the greatest curse to vivisectable animals” (French 1975: 68 & 415).^15^

Hoggan was a restrictionist who did not favour banning vivisection but rather felt that it needed to be “made amenable to public opinion” (Report 1876: 178). He believed the best way of making physiology answerable to the public was through concrete changes in the physical infrastructures of science. In order to operate as a boundary object linking scientists, students and the wider public, the anaesthetised animal had to be made visible—made *superliminal* as opposed to *subliminal*—in the infrastructures of textbooks and within laboratory buildings. These infrastructures, following Geoffrey Bowker and Susan Leigh Star’s (1999: 313–314) expansion of the boundary object concept, can be thought of as “boundary infrastructures”, namely structures institutionalized so that they are “sunk into the built environment … to keep things moving along.” In this case, what was needed for ‘cooperation without consensus’ on the question of vivisection were infrastructures that, in accordance with restrictionist demands for the laboratory animal to be made routinely visible to the public, enabled the surveillance and regulation of science, whilst also keeping science itself moving along, as opposed to hindering its practice.

As aforementioned, two important forms of scientific infrastructure that had raised antivivisectionist concerns had been textbooks and buildings. Both were feared to enable cruel experiments to be carried out. Textbooks were principally seen as dangerous on the grounds that they could be used by autodidacts and dilletantes as instruction manuals for the secret performance of vivisections in private quarters. Hoggan, like Anthony, was concerned by the potential expansion of private vivisection that textbooks allegedly allowed and recommended all such secret physiological experiments be banned, with imprisonment as a possible punishment for offenders (Report 1876: 178). But whilst the circulation of experimental instruction might threaten to open new spaces for vivisection outside of formal laboratories, textbooks in themselves were relatively easy to modify; the general mood at the Commission was that Klein and colleague’s *Handbook* was problematic because it failed to specify that anaesthesia should be induced for many of its experiments, but that this could be largely resolved through the addition of such specifications. The coupling of vivisection with anaesthesia could thus be made to travel relatively easily through textbooks as an infrastructure.

Buildings, however, were more difficult to make amenable to popular concerns, for they shielded experimenters from public view and, as property of individuals and institutions, were spaces in which the rights of occupants nominally trumped those of outsiders. Ritvo notes (1987: 145) that as late as 1895, the RSPCA were still complaining that the “doctrine of the sacredness of alleged rights of the citizen, the domicile, and of private property” continued to bar the organisation from access to claimed indoor sites of cruelty in private households, clubs and institutions. Buildings therefore needed to be made transparent to ensure that the coupling of vivisection with anaesthesia was travelling in the form of the anesthesised animal as a boundary object.

Bentham’s panopticon had been an influential earlier attempt to design a structure that minimised the ability of criminality to hide in the shadows of walls (Foucault 1995 [1975]). At the 1875 Commission, Hoggan suggested laboratory designs that were likewise panoptic. All permitted physiological experimentation “necessitating wounding or infliction of pain upon animals” should, he argued “be conducted in a suitable hall fitted with the necessary tables and apparatus… overlooked by a gallery, or galleries, into which the public could have unrestrained access by separate doors” (Report 1876: 179).

Hoggan held that anaesthesia, if “kept perfect”, was a means of making experimentation humane (Ibid: 178). However, he did not *trust* physiologists to always take the necessary care to prevent animal suffering without being monitored. Anaesthesia was not enough without public oversight, which required that infrastructures of concealment be transformed into infrastructures supportive of direct and virtual public “witnessing” (Shapin and Schaffer 2011: 60–65; see also Shmuely 2017: 44–45). Hoggan maintained that opening physiology labs to civic scrutiny would discourage “habitual or dilletante vivisectors”, but that “earnest workers for the good of humanity would not hesitate to work openly” (Report 1876: 178–179). These humane experimenters would moreover benefit from greater public access to their laboratories, for “The public mind and conscience accused at present of exaggerations would thus be afforded an opportunity of calming and rectifying itself, if no cruelty or other abuses were to be witnessed” (Ibid., p. 178). Transparency through the access of “publics” to scientific spaces would therefore be a win–win situation for those scientists who did ‘nothing wrong’.

However, Hoggan’s attempts to create new boundary infrastructures in the form of open laboratories was anathema to *laissez*-*faire* physiologists who viewed the suggestion that their work be open to scrutiny by lay members of the public as a threat to their autonomy and authority. “I think it would be most dangerous”, remarked William Turner, going further stating that “if physiological laboratories in this country were put under the inspection and supervision of persons of that kind we should become the laughing stock of scientific Europe” (Ibid: 163). John Simon, asked by Hutton for his thoughts on bringing the “conscience of the public” into the lab, affirmed he had no issue admitting “the thoroughly educated public” but was concerned that any such opening would also let in “the mob” (i.e. the lower classes) (Ibid: 75). Hoggan himself did not want to admit everyone. He rather suggested that “the number of persons in the gallery ought also to be limited, say to 10”, with “all admission therein to be by tickets issued gratis by the superintendent to all *respectable* persons above the age of 18 who might apply for them, *something like a reading ticket for the British Museum*” (Ibid: 178; our emphasis added). Clearly, he had in mind attendance being restricted to the political and educated elite. But it is important to note that this alternative would include most prominent British anti-vivisectionists, who in Simon’s view appeared increasingly to incite and become interchangeable with the ‘mob’. Hoggan’s suggestion was thus unacceptable to most supporters of physiology, and was not realised. But the issue of trust, as we will see, did not go away and did require an infrastucture for the anaesthesised animal to become a boundary object.

## Rebuilding trust: anaesthesia and legislation in a changing moral landscape

The 1875 Commission testimony of Emanuel Klein, co-author of the controversial 1873 *Handbook,* discredited the idea that scientists could be trusted to perform anaesthesia without supervision. Klein claimed to have “No regard at all” for animal pain and in his own experimental work “never use anæsthetics, where it is not necessary for convenience”. Further, when pressed as to what he meant by convenience, Klein defined it in terms of the experimenter’s desire not to be scratched or disturbed by the animal’s cries (Ibid: 183). Klein’s blunt disregard for animal suffering severely undercut the *laissez*-*faire* argument that British physiologists could simply be trusted to treat animals ethically.

Atalić and Fatović-Ferenčić (2009: 710–712) suggest Klein’s crudely candid performance may in part be put down to his being a foreigner who “did not know how to respond appropriately before the Commission”, and that his subsequent public image as a prototypical “monstrous vivisectionist” was also shaped by British xenophobia and anti-Semitism (Klein was an Austrian Jew). Regardless, there is no doubt that Klein’s testimony represented a turning point at the 1875 Royal Commission. French even implies that without Klein there may have been no 1876 Act, as “without the testimony of Emanuel Klein, several members of the Commission would have been entirely unwilling to sign a report recommending legislation of any kind” (1975: 103). This assessment is supported by Huxley’s private correspondence, first in an October 1875 letter to Darwin where he terms Klein “an unmitigated cynical brute” and confessed that “I would willingly agree to any law which would send him to the treadmill” (Huxley 1908: 171–172). Secondly, Huxley asserted in a May 1876 letter to Foster that “It is not Hutton who has beaten me, but Klein” (French 1975: 105).

Huxley thus admitted legislation necessary once it became clear that trust alone was not a sufficient infrastructure for holding science and the public together. Though he doubted the suitability of inspection, Huxley recognised that without external witnesses as mediators between science and public, there would not be trust. Moreover, there would be pressure to allow more intrusive observation regimes such as that suggested by Hoggan. It was preferable to have a set of government inspectors committed to ‘fair’ and ‘objective’ assessment overseeing licensing than to risk admitting the overtly hostile antivivisectionist mob into the laboratory. The present system of Home Office inspectors and licensing initiated by the 1876 Act was to be the ‘boundary infrastructure’ connecting scientists and the public in the context of this distrust.^16^

Building trust between science and the public centred both legislated supervision and anaesthesia as guarantors of humanity in the laboratory. Supervision, it should be stressed, was in many ways secondary to anaesthesia, for supervision had only become requisite because of the new technological possibilities anaesthesia offered. As the Committee noted, prior to the widespread availability of anaesthesia there was a need to “weigh in the one scale the infliction of great and perhaps protracted suffering, and in the other the sacrifice of knowledge, most important to mankind” (Report 1876: xiii). Without anaesthetics there was no possibility of performing many experiments without causing extreme animal pain. Scientists however could justify this suffering by appeal to the human betterment enabled by developing new experimental knowledge. Supervision in such a scenario had little value, as animals would suffer whether an audience was present or not. Further, the justification for such suffering would in any case be based on uncertain post hoc application of experimental knowledge. The choice for governments was therefore either to tolerate or ban vivisection, with the former being preferred in practice. Anaesthesia changed this by enabling experimentation with minimal pain, albeit dependent on technical proficiency and careful application. Scientists could therefore no longer argue that pain could be justified by the production of useful knowledge unless they could also conclusively demonstrate that anaesthesia could not be incorporated into the design of a necessary experiment.

Many scientists at the Royal Commission, moreover, appeared to recognise that anaesthetics had changed the moral landscape of experimentation by rendering a whole class of formerly excusable suffering as morally odious. Charles Darwin, on being asked for his view on “painful experiment without anæsthetics, when the same experiment could be made with anæsthetics”, responded that “It deserves detestation and abhorrence” (Report 1876: 234). Darwin spoke here not only on his own behalf, but also on the Committee’s, having been invited to the stand by Huxley with the express intention of countering Klein’s “mischief” (Huxley 1908: 172). The Committee ultimately concluded that Klein’s callousness could be countered by ensuring correct anaesthetic procedure was followed through a combination of proper training, licensing and inspections. This argument, however, was reliant on a set of unspoken claims about scientific sensitivity to animal pain, animal capacity to suffer, and the nature of anaesthesia itself, as we discuss next.

## (In)sensitivities and (un)certainties: the case of curare

At the time of the Commission, physiologists were already using curare in their experiments to a significant degree. A paralytic imported from the Amazonian rainforest, curare had become a popular means of immobilising experimental animals and was recommended for that purpose in Klein et al’s *Handbook*. It had also long been assumed to have anaesthetic properties but this had been questioned by Claude Bernard, who had shown that frog limbs, if isolated from the effects of curare on the rest of the body, still displayed sensitivity when pricked (Shmuely 2019: 7–9). Bernard also cited cases of human survivors of curare poisoning who had reported continuous consciousness whilst paralysed (curare, unlike ether or chloroform, was otherwise not widely used on human subjects) (Report 1876: 204–205).^17^ Hoggan took Bernard’s view on the matter, characterising curare as a “sham anæsthetic” utilised in preference to true anaesthetics primarily because it did not require an assistant to continuously administer, a “trouble… far too great for those who are in no way inconvenienced by the torture they may inflict” (Hoggan 1875: 526). In particular Hoggan singled out William Rutherford’s experiments on biliary secretion in dogs as “cruel in the extreme” due to their reliance on curare (Report 1876: 180). Hoggan clarified that “It would not be a cruel experiment under anæsthetics thoroughly applied” (Ibid: 201).

Hoggan’s protests over the use of curare contributed to its becoming one of the Commission’s greatest points of controversy. The unsettled nature of the science of curare was reflected in a substantial division amongst physiologists regarding its alleged anaesthetic properties—for example, Burdon-Sanderson and Foster, otherwise close allies, held divergent views on the matter (Shmuely 2019: 9–11). These debates do evidence that some physiologists were uncritical in accepting claims of curare’s anaesthetic efficacy. Emanuel Klein, when quizzed on the subject, largely deferred to Schiff’s authority on the matter (Report 1876: 188). Other physiologists supported curare on questionable theoretical grounds. George Henry Lewes, having opined curare to be “probably an absolute anæsthetic”, qualified that “I disbelieve entirely in sensation—I mean *conscious* sensation—without motion” (Ibid: 312). That curare had attained widespread use in British physiology without its anaesthetic qualities being fully ascertained does appear to support Hoggan’s claim that many physiologists were incautious or not fully considerate in their efforts to prevent animal pain. Whether cruelty was “deliberately inflicted”, as Hoggan charged, is more difficult to prove given the unsettled nature of curare science (Hoggan 1875: 526).^18^

The Committee ultimately recommended a moratorium on curare’s use without other anaesthetics, stating that “until the question shall be much better settled than it is at present this poison ought not to be regarded as an anaesthetic” (Report 1876: xix.).^19^ The 1876 Act (Section 4) then prohibited the use of curare as an anaesthetic. The debates over curare helped establish the need for strong scientific statement on whether a substance was effective in inducing unconsciousness and preventing pain before it could be licensed as an experimental anaesthetic. This in effect required that its powers be proven in both animals *and* humans. Curare had shown that without personal testimony, it was sometimes difficult to be certain, based on external appearances alone, whether a substance truly induced anaesthesia or merely paralysis. It thus troubled the claim that stillness in the supposedly anaesthetised animal was a guarantee of true obliviousness to pain.

But anaesthesia itself was a poorly understood condition attended by its own scientific and cultural uncertainties and anxieties. Even substances such as ether and chloroform whose anaesthetic properties were well documented through their widespread use on human patients could be difficult to interpret in their effects when applied to animals. Thus whilst the Commission sought to establish the anaesthetised animal as a boundary object around which cooperation could be built through interpretative flexibility, the liminal fuzziness of anaesthesia itself served to call into question the ‘arrangement’ or assemblage of experimentation that was being modelled by the anaesthetised animal – the very arrangement that allowed “different groups to work together without consensus” (Star 2010: 602) that is central to the boundary object.

## Anaesthesia between life and death: (de)stabilising the boundary object

Anaesthesia was generally met with a great deal of fear and anxiety as well as relief in Victorian Britain, due to uncertainties about the boundaries between pain and painlessness, consciousness and unconsciousness, recollection and forgetting. The physiological differences and communication barriers in human anaesthesia were exacerbated in animals, and this brought into question the extent to which the anaesthesised animal resolved the problem of pain. “I have tried myself”, the Oxford anatomist George Rolleston recounted at the 1875 Commission, “a number of experiments upon the action of anæsthetics, and I must say that it is not so easy a thing to know when you have an animal thoroughly anæsthetised”; indeed “the whole question of anæsthetising animals has an element of uncertainty about it” (Ibid: 68). Physiologists and their supporters sought to secure their claim that they were not causing unwanted pain by first equating anaesthesia with a deathlike state of oblivion. Second, they insisted on their overcautiousness in ensuring animals remained unconscious throughout the process, to the point of ensuring the dose of anaesthetic ultimately proved fatal.

Unlike cruelty, killing animals was broadly accepted in Victorian British society so long as it was for a suitable purpose—e.g., food, pest control or socially acceptable sport—and not unnecessarily painful. It was also viewed as a preferable alternative to even quite minor animal suffering. As Turner observes, dog shelters “paradoxically kill[ed] each year thousands of perfectly healthy animals… in order to spare them the cold, hunger, and possible starvation that befell the stray” (1980: 79). Killing animals for scientific research was also relatively uncontroversial and many alternatives to vivisection relied on it. Speaking at the Commission, Samuel Haughton, who opposed vivisection for demonstrative purposes, suggested other universities should follow the example of his own institution, Trinity College Dublin, where professors instead used “animals freshly killed, in which you can keep up artificial respiration” (Report 1876: 103). For Haughton, killing animals in itself was unproblematic—it was the problem of pain and potential for demoralisation that concerned him.

This relative cultural acceptance of animal death led some physiologists to draw an equivalence between death and anaesthesia. One such strategy involved invoking the “already dead” argument (Birke et al. 2007: 84–85).^20^ For example, William Sharpey maintained that “where anæsthetics are used it is simply a question of the sacrifice of an animal” (Report 1876: 23). On this basis he argued that “there need be no restriction imposed on experiments performed under anaesthetics, or in fact on experiments in which *the animal is rendered insensible in any appropriate way*” (Ibid; our emphasis). As means of inducing insensibility, Sharpey approved not only anaesthesia but also pithing (destruction of the brain or spinal column using a needle) and decapitation (Ibid). This conflation of anaesthesia with lethal techniques for inducing insensibility highlights how, like a precursor to Schrödinger’s cat, the anaesthetised animal was in many ways treated as both alive and dead. Indeed, Rolleston went as far as to argue that “If it is certain that the animal is under an anaesthetic, and if it is also understood… that the animal is put out of life before it returns to sensibility, the animal may then practically be considered as dead, albeit for physiological purposes it is a living machine” (Ibid: 66).

The construal of the anaesthetised animal as a boundary object between life and death was also supported by a widespread contemporary belief in anaesthesia as a state intermediary between life and death. As Pernick has argued, anaesthesia was only one of a number of 18th and 19th century scientific discoveries that “combined to render the boundary between life and death frighteningly indistinct” (Pernick 1988: 20). Thus anaesthesia possessed a liminal uncanniness, being, in the words of renowned American anaesthesiologist Julian Chisolm, a “borderland in which life in many respects so simulates death” (Chisolm 1888: 6). One important way anaesthesia simulated death, argued its supporters at the Royal Commission, was that like death it “destroys consciousness” (see Report 1876: 38, 103 and 123 for Foster, Haughton and Pritchard’s affirmations of this claim). This power of anaesthesia to induce a deathlike oblivion was widely accepted. Even a critical supporter of vivisection such as Haughton admitted that a chloroformed frog was no more capable of feeling than a pithed one. He qualified, however, that “if you did not mutilate the frog to which you applied the anæsthetic, you might let it live again and enjoy a few more months of life. If you pith it you destroy it” (Ibid: 103).

Recovery from anaesthesia was seen as risking animal suffering and many physiologists sought to avoid this by overdosing the animal. This met with the approval of humanitarians such as the RSPCA’s Secretary John Colam, who was pleased to see that the scientists at a lab he had inspected did not just euthanise the animals post-experiment but moreover had first administered a dose of chloral “so heavy that I have not the slightest doubt they could not have recovered from it” (Ibid: 81). Overdosing anaesthesia not only pre-emptively prevented intraoperative recovery but also helped close the gap between anaesthesia and death through eliminating the possibility of revival, thus helping secure the anaesthetised animal’s ethical status as ‘already dead’.

This fatal deployment of anaesthetics did not meet with universal acceptance among physiologists. The surgeon Robert McDonnell, on being asked how long he kept the dogs subject to his experiments on arterial torsion alive, responded that whilst “The pain of the operation is severe”, that experienced afterwards was “so almost nil that it would be humane in fact to allow the animal to recover altogether”. Affirming that he thought euthanasia would be “a cruel sacrifice of life”, McDonnell elaborated that “We do not kill dogs to save them from such pain as that” (Ibid: 229). McDonnell here challenged Sharpey’s claim that the anaesthetised animal was ‘already dead’ by arguing that a dog might have future interests that outweighed its interest in avoiding postoperative pain, which euthanasia would curtail. John Simon, whilst supporting vivisection, had similar reservations about dismissing animal interests in future life. He maintained that scientists should be “anxious not all to underrate the real fact that the life of the animal is sacrificed for physiology… and frankly to accept as our responsibility that physiology does involve a limited sacrifice of animal life” (Ibid: 74).

The most vocal objection to killing anaesthetised animals came from the Oxford anatomist Henry Wentworth Acland:

I am not at all prepared to say that I think that even experiments causing painless death needlessly ought to be performed… I know some persons think that it is a matter of entire indifference to take away the life of an animal. I do not think so; I should act in a way which would do great injury to my conscience if I were needlessly to put to death the meanest animal; I could not do it. And, therefore, I cannot say that because I can put an animal to death without pain, therefore I may unnecessarily destroy an animal, in order to show a thing which I can show just as well upon a diagram (Ibid: 42).

McDonnell, Simon and Acland had in common the belief that vivisection played an important role in scientific investigation. But all three also saw animal death as problematic unless necessitated by postoperative pain or justified by the knowledge gained. The Committee tacitly acknowledged these criticisms, affirming that where “the injury inflicted would be very small and the pain would be almost nil”, it would be “more humane to permit the animal to enjoy life, than to destroy it” (Ibid: xx). In practice, however, euthanasia became effectively mandated by the 1876 Act, which established that “The animal must, if the pain is likely to continue after the effect of the anæsthetic has ceased, or if any serious injury has been inflicted on the animal, be killed before it recovers from the influence of the anæsthetic” (1876 Act: Section 3).

Our analysis suggests this mandating of death was a necessary part of consolidating the anaesthetised animal as a boundary object. Hoggan, Jesse and other antivivisectionists had insisted anaesthetics were unreliable for preventing animal pain and were not relied upon by physiologists. By aligning anaesthesia with deathlike oblivion, instructing experimenters to kill animals before they could awaken, and promoting overdoses of anaesthetics, leading British scientists and humanitarians were able counter major antivivisectionist concerns. Proponents of vivisection were here able to draw upon a much greater cultural acceptance of animal death than of suffering. Mandating death, however, also bypassed the issue of animal interests in life. As McDonnell suggested, animal interests in continued life might well outweigh the pain of carrying surgical injuries. Whilst the legislation gave some leeway to ‘allow to live’ and also included certificates granting investigators a right to allow recovery even if the animal would suffer pain, its general effect was to promote an increased application of euthanasia for humane purposes.

## Conclusion: the anaesthetised animal as ‘boundary object’

We have posited that the anaesthetised animal was a ‘boundary object’ across the worlds of experimental physiologists, anti-vivisectionists and legislators, thus holding together temporarily the humanitarian and scientific quarters of the British ruling class. The idea of a boundary object developed, in part, from Susan Leigh Star’s conversations with Anselm Strauss, and his notion of society as an assemblage of social worlds and arenas: sustained areas of interest or concern that bring different groups of people together over time (Strauss 1978). Within this context, Star and Griesemer (1989) framed boundary objects as those things that different social worlds use, things that must travel between and are taken up by different social worlds as they engage in their key activities. These things need to be both robust enough to serve different purposes across diverse social worlds, while also being flexible enough to allow particular social worlds to proceed with their own work. Boundary objects are hence key sites for exploring power relationships. Furthermore, they are key sites for the development of standards, as the compromises concretised in boundary objects require formalisation to be made durable across different social worlds (Griesemer 2015). Standardisation was, as we have seen, a central concern of the Commission in its efforts to determine the best means of ensuring the proper application of anaesthetics in physiology experiments. Star later developed the idea of boundary objects through her work with Geoffrey Bowker on infrastructures (Bowker and Star 1999), which are larger, more sedimented and thus often invisible sites of connecting social worlds. For the anaesthetised animal to be a boundary object, the social world of regulators and the infrastructures of textbooks and inspections had to be developed in tandem.

Star’s (1983, 1985, 1989) own research on efforts to map the brain from 1870 onward explored the theme of anti-vivisection in the UK during the 19th century. It focused particularly on the ways in which scientists united in opposition to a ‘common enemy’ at this time: anti-vivisectionists (Star 1985: 395). In fact, Star’s research included key figures involved in the 1875 Commission. For Star (1985), the slow rise of physiology in Britain, and the restrictions put in place by the 1876 Cruelty to Animals Act, created conditions of taxonomic, technical, as well as political uncertainty at the laboratory level. These uncertainties required a kind of management by scientists. Star’s goal was to make this work visible, as part and parcel of creating global scientific knowledge and certainty.

Star’s key concern was to map how these local uncertainties were translated into global certainty or disciplinary knowledge in this context. Our analysis has troubled a key boundary present in Star’s analysis: pro-vivisectionist/scientists versus anti-vivisectionist. We have shown how the anaesthetised animal was not simply a compromise between these two poles but also a compromise within science itself. Boundary objects are means to create cooperative working conditions in an arena where different social worlds and subworlds are making competing claims. The interpretive flexibility of boundary objects allows conflicting imaginaries to operate in the same space (Star 2010). Sometimes, this plasticity enables groups with contradictory interpretations to work alongside or past each other. Star (1993) wrote of this as cooperation without consensus. We have argued that at the 1875 Commission the anaesthetised animal, as a boundary object around and through which different parties could operate, was such a site of compromise.

Making the anaesthetised animal the boundary object that was central to the Commission’s debates helped to reinforce the bonds holding together the social world of British science. But this was achieved in part by imposing an internal boundary within British science between the ‘moral scientists’ that made up the majority of the social world and immoral ‘mavericks’ such as Klein who formed a subworld of science. Civil society was called upon to police the composition of the social world of science, through legislative regulation and inspection of physiology laboratories. Regulators and legislators were thus made part of vivisection as an arena. This invited interposition of civil law and its enforcers into the arena of vivisection thus reflected a breakdown of trust engendered by the vivisection controversy not only between scientists and other members of the British ruling class but also *within* the British scientific community. Making the anaesthetised animal into a boundary object kept the social world of science together, specifically an older generation uncomfortable with animal experimentation and younger, more professionalised scientists seeking to import physiological techniques based on vivisection from France and Germany. Assuaging concerns within the scientific social world therefore required the development of supporting “infrastructures” that would contribute towards standardising ethical laboratory practices and thereby help consolidate the anaesthetised animal as a boundary object. This included laws, licenses, inspection regimes and reforms to textbooks and pedagogy, in order to help ensure anaesthesia was properly applied where required. As Shmuely (2017) has shown, these tools and frameworks not only attempted to compensate for the loss of implicit public trust in scientists as respectable members of the gentlemanly elite but also contributed to shaping internal standards within the social world of British science regarding what constituted ‘ethical vivisection’. This complicates the narratives of polarisation between ‘science and society’ present in much of the secondary literature.

Diverse positions on the question of animal death further highlight how the 1875 Commission was not simply ‘physiologists versus antivivisectionists’—it was also a debate *within* the British scientific social world about what constituted morality in science and which interests mattered. Anaesthetics could be constituted as conducive to animal welfare only through negotiating specific parameters of concern, centred around removing pain whilst largely excluding consideration of animal interest in post-operative life. The Commission’s alignment of anaesthesia with death, by treating it as a deathlike state, was considered broadly acceptable and went on to influence the 1876 Act’s mandating of death as a necessary endpoint to vivisection if anaesthesia was to be regarded as fully effective. This judgement has heavily influenced the conduct of British animal experimentation, so that until recently there has been a pervasive consensus that humanely concluding a physiology experiment necessitates euthanising the experimental animal. This legally enforced common code of practice may, we suggest, have at times obscured the still extant question of whether it might not be “more humane to permit the animal to enjoy life, than to destroy it”.^21^

## Footnotes

1 Experiments also needed to be “performed with a view to the advancement by new discovery of physiological knowledge or of knowledge which will be useful for saving or prolonging life or alleviating suffering”, entailing that the ‘object of research’ could not be trivial or lack utility. There were, however, loopholes through which animal pain was permitted to persist. Experiments could be made without anaesthetics and animals could be recovered from anaesthesia in a painful state by licence holders alone, and only if this would compromise the experiments (1876 Act, Clause 3).

2 In using the word consensus, we do not intend to convey the idea of full agreement that is the conventional understanding of the term. Rather, we use consensus through Star’s (1993) idea that boundary objects are sites where there can be cooperation without consensus. While temporary, at the 1875 Commission the construction of the anaesthetised animal as a boundary object meant that people with different perspectives were able to have a dialogue and cooperate in a manner that created a short lived consensus where tradeoffs were made to create an outcome that many found at least tolerable.

3 By ‘humanitarians’, we refer to the broader set of British movements concerned with animal welfare, and more generally with societal suffering. Antivivisectionists were a subset of these movements but far from the most influential of those involved at the 1875 Royal Commission. The RSPCA, the most prominent of the British animal welfare organisations, whilst pivotal in helping bring about the Commission, had since the 1860s held the official position that whilst avoidable animal pain was undesirable cruelty, vivisection in itself was permissible so long as carried out under anaesthesia (French 1975: 32–33). Many physiologists, moreover, whilst regarding it necessary where possible to be humane to experimental animals, believed there to be no contradiction in describing themselves as pursuing humanitarian ends whilst sacrificing ‘lower beings’ for the sake of medical research. We acknowledge that ‘humanitarian’ and ‘humanity’ were thereby complex and contested categories at the Royal Commission but believe ‘humanitarian’ nevertheless a good general description of those groups expressing concern about animal welfare and pain versus those groups more concerned with the interests of advancing scientific knowledge and technique.

4 Antivivisectionists would subsequently sometimes attack the class credentials of vivisectors, as seen in Frances Power Cobbe’s assertion that most British doctors were “sons of men of the secondary professional classes or of tradesmen”, and were thus “a *parvenu* profession, with all the merits and the defects of the class” (French 1975: 341). It should be noted that the opening up of science to middle class professionals was not a socially isolated process, being part of a broader mid-19th century realignment of the British class system, other manifestations of which included the extension of the franchise in 1832 and 1867 to incorporate middle class men with smaller property holdings and ‘respectable’ working men. Lester (2001: 138–175) ties this lowering of social barriers within the propertied and entrepreneurial classes to anxiety about working class mobilisation and humanitarian disillusion with efforts to ‘civilise’ the poor at home and non-whites in the empire. Cobbe’s attack on ‘*parvenu*’ doctors was reflective of a reverence common among antivivisectionists for an older humanitarian tradition, tied to aristocratic notions of *noblesse oblige*, which middle class professionals were perceived as rejecting in favour of their own acquisitive advancement.

5 See also Oster (1989: 174), for Boyle’s similar misgivings about vivisection: “euident it is, that a Feeling they haue of Paine; which causelesly to make them endure, is a thing manifestly contrary to humanity”.

6 See Tague (2015), which explores the rise of petkeeping in 18th century Britain to track how changing notions of animals were entangled with the changes brought about through British imperialism and wealth at that time.

7 1835 Act Section XXI. ‘Construction of Terms used in this Act’: “where the Word "Cattle" is used alone in this Act the same shall be understood and taken for any Horse, Mare, Gelding, Bull, Ox, Cow, Heifer, Steer, Calf, Mule, Ass, Sheep, or Lamb, or any other Cattle or domestic Animal”.

8 See Section 29 of the 1849 Act: “The word “animal” shall be taken to mean any horse, mare, gelding, bull, ox, cow, heifer, steer, calf, mule, ass, sheep, lamb, hog, pig, sow, goat, dog, cat, or *any other domestic animal*” (our emphasis). Blood sports involving the capture of wild beasts for baiting or running were the main exception to this restriction to the domestic, but there was also no provision for wild animals captured for non-sporting purposes or for wild animals targeted by pursuit blood sports, shooting or hunting. The definition of ‘wild’, moreover, in practice included semi-domesticated animals such as gamebirds, rabbits and deer, and was applied largely on a species, not individual level—a tame individual from a generally wild species would not be accorded the privileges granted to traditional domestic species.

9 Cobbe had in 1863 whilst living in Florence organised protests within the English colony against the physiologist Moritz Schiff. The RSPCA itself had in the late 1850s and early 1860s campaigned against the ‘Alfort system’ in French veterinary schools, whereby students performed unanaesthetised surgical operations on knackered horses to acquire manual dexterity. See French (1975: 30–31 & 63).

10 For details on the RSPCA’s position, see French (1975: 32–33).

11 Cobbe later protested that “The Royal Commission was so constituted as to afford every advantage of fair play to the physiologists”, asserting there to have been two *experts* on the side of science [Huxley and Erichsen]; and the progress of the inquiry shows that these two gentlemen acted throughout as counsel, the one for the physiologists and the other for the medical profession” (Cobbe 1877: 340).

12 See Report, pp. 2, 13, 43, 50 for use of the language of ‘necessary experiment(s)’. The use of ‘unnecessary experiment(s)’ as a contrast category is also common throughout the Report.

13 See Arthur de Noé Walker’s protestation (Report 1876: 243) of the alleged “liberal supply” of animals offered to David Ferrier. Walker upheld that “that the number allowed to each licensed vivisector should be limited” to a ‘sufficient supply’ necessary for the purposes of carrying out a particular experiment.

14 See also Hoggan’s explanatory comment that “the public have generally supposed that anæsthetics were used”, and consequently “did not feel called upon to make any demonstration… while the animals were suffering pain all the time” (Report 1876: 203).

15 Hoggan is identified by French (1975: 302) as “More than anyone else… responsible for antivivisectionist cynicism about anesthetics”.

16 Shmuely (2017) provides a very useful account of the workings of the Act and the Inspectorate between 1876 and 1913, when the Act was revised following the Second Royal Commission on Vivisection. She likewise puts particular stress on the role of infrastructures and legislation in supporting the mandating of anaesthesia and combatting the trust gap between physiologists and the general public, noting the particular importance of paperwork and legislative consultation in resolving problematic cases where possible breaches of the Act occurred or could potentially take place—“The cracks that threatened to break the illusion of a painless animal were quickly filled with forms, conditions, and legal opinions” (p. 38).”.

17 Further evidence was presented at the Committee by Arthur Gamgee, who had conducted experiments using small doses of curare on epileptic children in an effort to determine if it had any beneficial effect on their condition. He reported that where a strong enough dose was administered to induce transient paralysis, an otherwise “peculiarly stolid and unexcitable child became obviously frightened”. Gamgee concluded that “in this little patient I was able to determine very decidedly that sensibility was not at all impaired” (Report 1876: 262).

18 Stewart Richards does, however, suggest that Rutherford, whose work Hoggan condemned, was in “full knowledge that [curare] induced motor paralysis only”, having adopted its use “quite overtly to prevent 'the disturbing effect of irregular muscular movement upon the biliary flow’” (true anaesthetics by contrast did not offer “a reliable control value”) (Richards 1986: 204).

19 This recommendation was in spite of Huxley’s support for those physiologists using curare and his view that Bernard had “‘jumped to the conclusion” when all he had shown was that curare disturbed the reflex action (Shmuely 2019: 11).

20 See also Arluke (2004), which details his ethnographic work with medical students attending ‘dog lab’. Examples of the ‘already dead’ rationale identified by Arluke include the argument that the animal is already ‘marked for death’ as an unadopted stray, that it is already under heavy anaesthesia when the students enter the room so is not conscious and will feel not pain, and will ultimately be killed at the end of the demonstration.

21 For example, current day questions about whether or not a 4th R of rehabilitation should be added to the 3Rs—refine, reduce and replace animals in research—articulates and challenges the presumption that animals do not have an interest in further life (Pereira and Tettamanti 2005).
